# Editorial: Interorgan crosstalk mediated by exerkines: the role of exercise in health and disease

**DOI:** 10.3389/fendo.2024.1459020

**Published:** 2024-08-05

**Authors:** Joanna Jaworska, Katarzyna Micielska, Martina Faraldi

**Affiliations:** ^1^ Department of Physiology, Medical University of Gdansk, Gdansk, Poland; ^2^ Department of Physical Education and Lifelong Sports, Poznań University of Physical Education, Poznań, Poland; ^3^ Laboratory of Experimental Biochemistry and Molecular Biology, IRCCS Istituto Ortopedico Galeazzi, Milan, Italy

**Keywords:** exercise, exerkines, tumor growth, irisin, extracellular vesicles, metabolic diseases, hippocampal neurogenesis

In the last two decades, humoral factors secreted into circulation by different organs in response to acute exercise or regular training have gained significant attention. The term “exerkine” was introduced in 2016 (1), although the concept of humoral factors mediating the benefits of exercise has been recognized for a long time. Exerkines are increasingly acknowledged to include a broad range of signaling molecules, such as cytokines, nucleic acids (microRNA, mRNA, and mitochondrial DNA), lipids, and metabolites, which are often driven by cell-specific extracellular vesicle secretion (2). These molecules play a potential role in driving the well-established benefits of exercise, such as preventing and mitigating disease, promoting health, and increasing resilience. This Research Topic collected four publications, namely one original research article, one review, and two mini reviews, all of which have enhanced the understanding of the complex organ crosstalk related to exercise. These contributions are crucial for formulating specific preventive or therapeutic plans.

Obesity rates are increasing year over year, leading to metabolic diseases. Exercise plays a crucial role in mitigating obesity-related complications, promoting negative energy balance through increased energy expenditure, and influencing appetite-regulating hormones to help control hunger, satiety, and body weight. Therefore, Li et al. performed a cross-design study, in which they recruited 14 obese adults and investigated the effects of combining moderate-intensity continuous exercise (MICE) with blood flow restriction (BFR) on N-lactoylphenylalanine (Lac-Phe) and appetite regulation in obese adults. Lac-Phe is a new type of exerkine closely associated with lactate, which has potential appetite-inhibiting effects. Therefore, the authors decided to divide the subjects not based solely on the intensity of continuous exercise (60% VO2max, 200 kJ for both exercise groups), but also on the application of BFR. BFR can induce local tissue hypoxia and increase lactate accumulation. Interestingly, the authors of the study demonstrated that a single session of combined MICE and BFR exercise reduced the appetite of obese adults by promoting the secretion of Lac-Phe and ghrelin. This suggests that additional hypoxia, achieved by limiting blood flow during exercise, may be a key element in the fight against obesity.

In their review, Kraemer and Kraemer also highlighted the importance of ghrelin in various responses to physical exercise, as well as its role in growth hormone (GH) - insulin-like growth factor-1 (IGF-1) signaling. Thus, ghrelin, produced and secreted in the stomach, can be modulated by physical exercise. It not only affects the feeling of hunger but also potentially indirectly participates in neurogenesis. Moreover, the authors point out how different forms of exercise influence the peripheral production of specific endocrine factors, with particular emphasis on brain-derived neurotrophic factor (BDNF), estrogen, testosterone, irisin, vascular endothelial growth factor, erythropoietin, and cortisol. Additionally, they describe mechanisms through which these endocrine responses to exercise induce cellular changes that increase hippocampal neurogenesis and improve cognitive function.

Irisin is another exerkine that has an impact on brain health and cognition, stimulating the increase of BDNF expression. Trettel et al. widely reviewed irisin significance in maintaining whole-body homeostasis in response to exercise, throughout showing different interorgan crosstalk and dependencies. They also emphasize irisin’s role on inflammation status upregulation through reduction of pro-inflammatory cytokines and reactive oxygen/nitrogen species (ROS/RNS). However, knowing that irisin is mainly produced by skeletal muscle during contraction, the authors drew attention to the appropriate selection of training sessions and programs, based on intensity, duration, and exercise type for different populations. Their findings support the hypothesis that long-term health benefits are mediated by optimal irisin concentration, achieved in response to regular exercising.

While physical activity is well-recognized for its relevance in the prevention and treatment of non-communicable diseases, there is also evidence supporting its importance in cancer and malignancies. Siqueira et al. summarized the possible beneficial impact of exercise-induced extracellular and particles vesicles (EVPs) cargo on oncologic outcomes. The authors emphasized the meaning of EVPs cargo, such as miR-150-5p, miR-124, and miR-486, and its upregulation in response to exercise, on tumor growth and metastasis, and on the immune system and body composition changes in cancer. Presented results describe available research conducted with humans and on animal models, comparing both the effect of a single training unit and a whole training program.

The presented Research Topic provides support for the prominence of different exerkines as beneficial-change mediators during exercise, specifying their role as novel preventive/therapeutic agents in different diseases.

## Author contributions

JJ: Conceptualization, Writing – original draft, Writing – review & editing. KM: Writing – original draft, Writing – review & editing. MF: Writing – original draft, Writing – review & editing.
